# Neiguan (PC6)-based acupuncture pretreatment for myocardial ischemia reperfusion injury: A protocol for preclinical systematic review and meta-analysis

**DOI:** 10.1097/MD.0000000000020792

**Published:** 2020-07-10

**Authors:** Qiqi Yang, Huifang Mao, Xia Chen, Yanji Zhang, Xiaolei Zhang, Zhenzhen Liu, Gongdao Jiang, Wei Huang

**Affiliations:** a Hubei University of Chinese Medicine/The Co-innovation Center for Preventive Treatment of Disease of Acupuncture-moxibustion in Hubei Province; b Hubei Provincial Hospital of Traditional Chinese Medicine, Wuhan, China.

**Keywords:** acupuncture, myocardial ischemia reperfusion injury, Neiguan (PC6), pretreatment, protocol, systematic review

## Abstract

**Background:**

Myocardial ischemia reperfusion injury (MIRI) is 1 of the leading causes of disability and mortality worldwide in the cardiovascular diseases. Acupuncture has been widely applied in the treatment and prevention of cardiovascular diseases in recent years. This systematic review protocol aims to provide the methods for evaluating the efficacy of Neiguan (PC6)-based acupuncture pretreatment in animal models of MIRI.

**Methods and analysis:**

The electronic databases of PubMed, Embase, Cochrane Library, as well as the Chinese databases such as China National Knowledge Infrastructure, Chinese Science and Technology Periodical Database, China Biology Medicine Database and WanFang Database will be searched from inception to November 2019. The outcome measures were myocardial infarct size, the level of ST-segment elevation, left ventricular ejection fraction, shortening fraction, arrhythmia score, cardiac enzymes, and cardiac troponin. Study inclusion, data extraction and quality assessment will be performed independently by 2 reviewers. RevMan 5.3 software will be used for the data synthesis and the quality of each study will be assessed independently by using the Collaborative Approach To Meta-Analysis And Review Of Animal Data From Experimental Studies checklist with minor modification.

**Results:**

This review will provide a high-quality synthesis of Neiguan (PC6)-based acupuncture pretreatment for MIRI in animal models

**Conclusions:**

This systematic review will provide conclusive evidence for whether Neiguan (PC6)-based acupuncture pretreatment is an effective intervention in animal models of MIRI.

**Trial registration number:**

PROSPERO CRD42020175144.

## 1 Introduction

Acute myocardial infarction (AMI) is the myocardial necrosis caused by acute ischemia and hypoxia of coronary artery, which is 1 of the leading causes of disability and mortality worldwide.^[1,2]^ In the US, the overall prevalence for AMI is 3.0% in adults older than 20.^[3]^ Within China, the mortality rate of AMI in urban areas and rural areas is about 58.69/100,000 people and 74.72/10000 people respectively,^[4]^ and it is estimated that the total annual cost of hospitalization for AMI alone is up to RMB 19.85 billion.^[5]^ Although various timely and effective revascularization, such as percutaneous coronary intervention and coronary artery bypass grafting, have been widely used in clinic to rescue ischemic myocardium, and can save patients’ lives to a certain extent.^[6]^ However, the process of reperfusion may further damage myocardial ultrastructure, metabolism and function, even lead to a further death of cardiomyocytes which contributes up to 50% of final myocardial damage.^[7,8]^ Moreover, it has been reported that nearly 900000 people have cardiovascular events caused by myocardial ischemia reperfusion injury (MIRI) such as perioperative myocardial infarction, heart failure and sudden cardiac death every year in the world, with a mortality rate of 10% to 15%.^[9]^ All in all, MIRI not only reduce the benefits of reperfusion therapies, lead to a significant increase in the economic burden of MIRI-related medical systems, but also seriously threatens patients’ lives.^[7]^

At present, prevention plays an important role in controlling MIRI. It has been proved that pretreatment before ischemia or reperfusion can make body generate moderate pressure, and active the endogenous adaptation mechanism to prevent or reduce the damage of subsequent diseases.^[10]^ The current means to activate the endogenous protection mechanism of body mainly include ischemic preconditioning,^[11]^ drug preconditioning,^[12]^ temperature preconditioning^[13]^ and exercise preconditioning,^[14]^ which can enhance the tolerance of the heart to the damage caused by myocardial I/R injury. However, these pretreatment measures are cumbersome to implement and inevitably cause trauma to the body, thereby greatly limiting their translation into clinical benefits. Therefore, seeking an effective, non- invasive and simple pretreatment method has become an urgent clinical need.

Acupuncture, as a key component of traditional Chinese medicine (TCM), is a therapy that uses a sterile needle to penetrate a specific acupoint in body to treat specific diseases.^[15]^ With minimal side effects and strong operability, it has been 1 of the most widely used complementary therapies in many countries.^[16]^ Previous studies have shown that acupuncture pretreatment can also activate endogenous protective mechanisms of body and improve immunity.^[17]^ In addition, the significance of acupoint selection in acupuncture therapy has also been affirmed. Neiguan (PC6) acupoint is the Luo-connecting point of pericardial meridian, which has the specific connection with the heart. Acupuncture at Neiguan (PC6) has dual-directional regulation effects on cardiovascular system, which has the characteristics of specific treatment of cardiovascular disease.^[18]^ A large number of studies have revealed that Neiguan (PC6)-based acupuncture pretreatment can exert cardio-protective effects in animal models of MIRI by improving coronary vasodilation and energy metabolism, inhibiting of oxidative stress, inflammatory response and apoptosis.^[19–22]^ Therefore, Neiguan (PC6)-based acupuncture pretreatment undoubtedly provides new inspiration for the clinical prevention and treatment of MIRI.

However, due to low quality,small samples of included studies and no study protocols reported in advance, 2 systematic reviews published in Chinese have failed to translate the efficacy of acupuncture into clinical benefits.^[23,24]^ And 1 review mainly focused on the add-on effect of electroacupuncture and ignored the critical effect of preconditioning in MIRI.^[24]^ Meanwhile, considerable new studies in this field have been published within past years. Thus, it is necessary to make a systematic review and meta-analysis based on the most comprehensive and up-to-date resources to provide a convincing conclusion whether Neiguan (PC6)-based acupuncture pretreatment is an effective cardio-protective intervention for MIRI.

## 2 Methods

### 2.1 Study registration

This systematic review protocol has been registered on PROSPERO with number CRD42020175144 (https://www.crd.york.ac.uk/prospero/display_record.php?RecordID=175144). Additionally, the current protocol report adheres to the Cochrane Handbook for Systematic Reviews and Meta-Analysis Protocol guidelines.^[25]^ Any change of the review will be described if needed.

### 2.2 Inclusion criteria

#### 2.2.1 Types of studies

Only randomized controlled animal study published in English and Chinese about acupuncture for MIRI will be included.

#### 2.2.2 Types of participants

Rat models of MIRI which were induced by ligation of the left anterior descending coronary artery (LAD) regardless of their age and sex.

#### 2.2.3 Types of interventions

Only manual acupuncture or electroacupuncture (EA) at Neiguan (PC6) point alone or a combination of PC6 and other acupoints will be used in experimental group. The intervention time is limited to before reperfusion.

#### 2.2.4 Types of comparators

Control intervention will be limited to no treatment or sham acupuncture.

#### 2.2.5 Types of outcome measures

Myocardial infarct size, the level of ST-segment elvation, left ventricular ejection fraction (LVEF), shortening fraction (FS) or arrhythmia score will be evaluated as the primary outcomes. And the secondary outcomes were cardiac enzymes (creatine kinase, creatine kinase-MB, lactate dehydrogenase, etc) or cardiac troponin (cardiac troponin T, cardiac troponin I, cardiac troponin C).

### 2.3 Exclusion criteria

(1) Case report or cross-over studies or review or meta-analysis or meeting abstract.
(2) Non-rat animal model or in vitro animal model or in silico models.
(3) MIRI animals with complications or induced by modeling methods other than ligation of LAD.
(4) Acupuncture therapies combined with other forms of acupuncture (such as auricular acupuncture, acupoint injection, moxibustion and etc) or TCM.
(5) Other acupuncture treatments were used in control group.
(6) Duplicate publication or literature published by identical data.
(7) Not published in peer-review journals.
(8) No predetermined outcome index.

### 2.4 Search methods for identification of studies

#### 2.4.1 Electronic searches

Experimental studies estimating the efficacy of Neiguan (PC6)-based acupuncture pretreatment in animal models of MIRI were systematically searched from EMBASE, PubMed, Cochrane Library, Wanfang database, China National Knowledge Infrastructure, Chinese Science and Technology Periodical Database, China Biology Medicine disc from inception to November 2019. No restrictions on language and publication were applied in the search strategy. Moreover, reference lists of potential studies were searched for relevant studies. And the searches will be re-run just before the final analyses to retrieve the most recent studies eligible for inclusion.

#### 2.4.2 Searching strategy

The search strategy for PubMed is shown in Table 1, which includes all search terms. Other electronic databases will be used the same strategy.

**Table 1:**
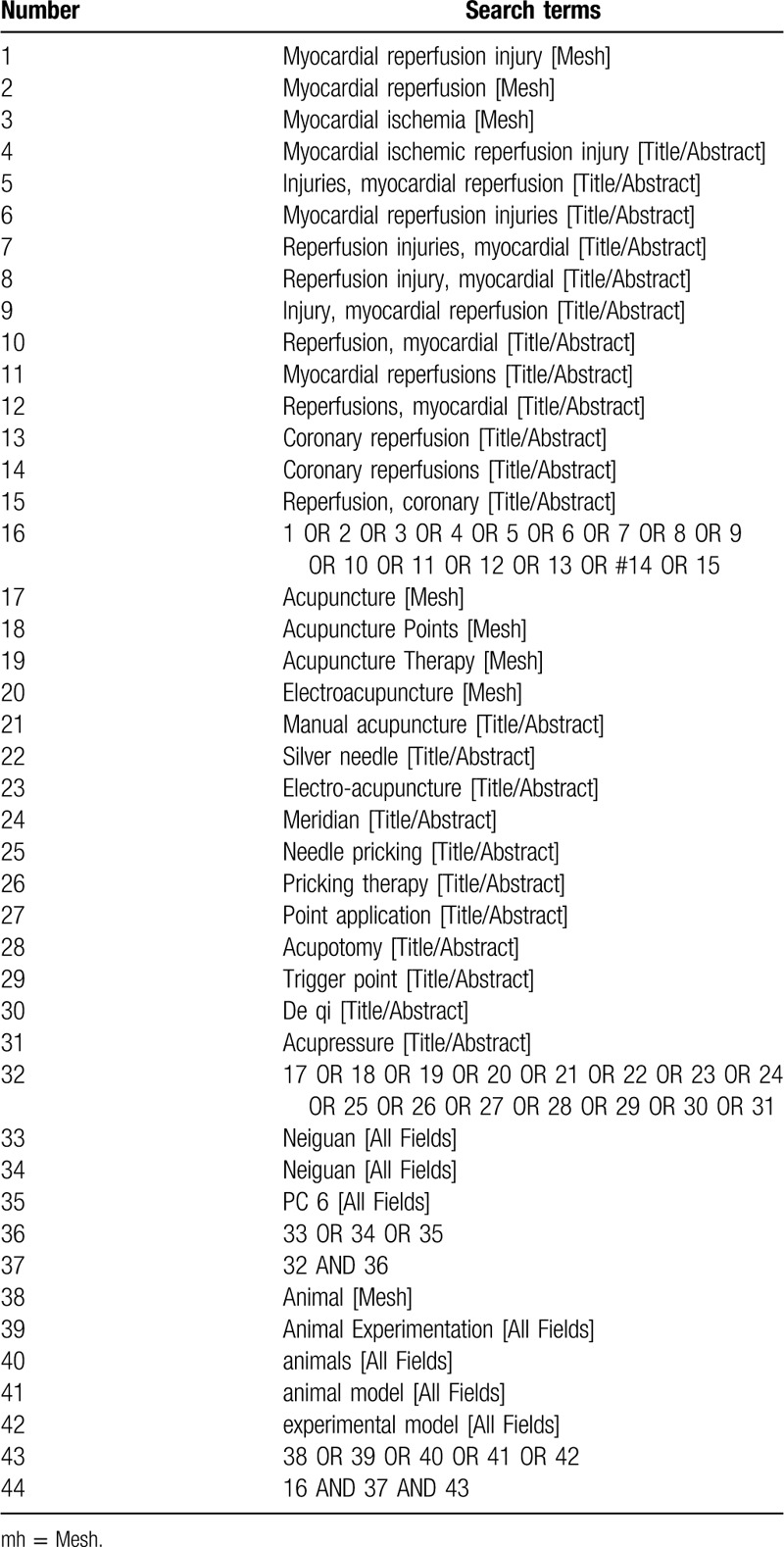
Search strategy for PubMed database.

### 2.5 Data collection and analysis

#### 2.5.1 Selection of studies

**Figure 1:**
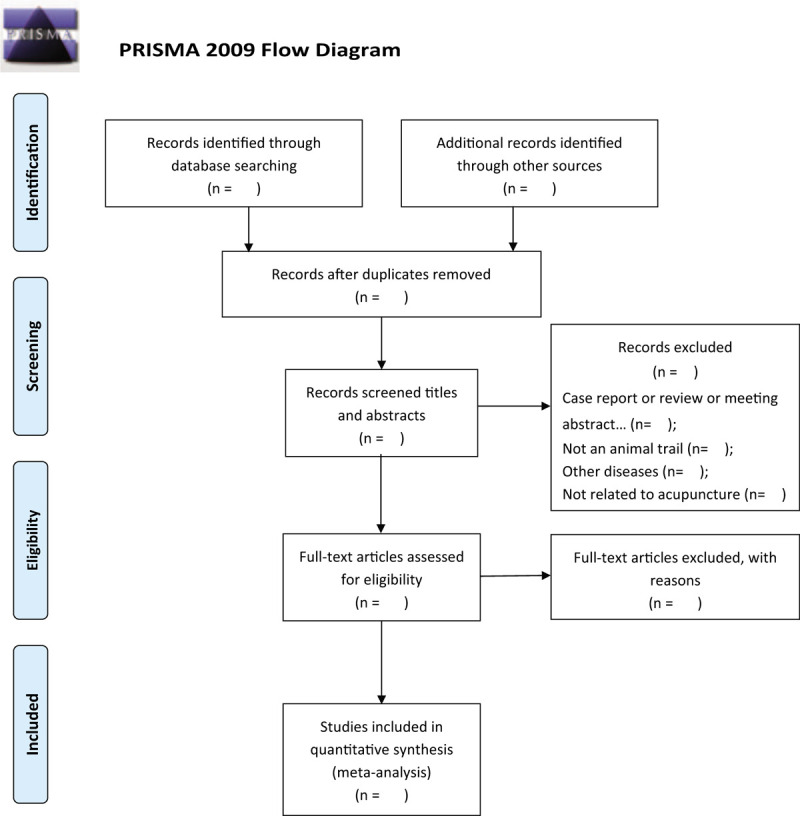
Flow diagram of the study selection process.

All reviewers will receive professional training to understand the objective and process of the review before the selection of studies. All the retrieved studies will be managed with EndnoteX7, and the duplicated studies will be discarded. Two review authors (YQQ and ZYJ) screened independently titles and abstracts of studies to identify studies that potentially meet the inclusion criteria outlined above. Then the full text of these potentially eligible studies will be independently assessed for eligibility by 2 reviewers (YQQ and ZYJ). Any disagreement between them over the eligibility of studies will be discussed with the third reviewer (ZXL). The procedures of study selection will be performed in accordance with the Preferred Reporting Items for Systematic reviews and Meta-Analysis flow chart (see Fig. 1).

#### 2.5.2 Data extraction and management

Two independent authors (ZXL and LZZ) extracted the following details from included studies:

(1) name of first author, year of publication;
(2) the detail information of animals for each study, including animal species, number, sex, and weight;
(3) the use of anesthesia in the experiment and the methods to establish animal models;
(4) information on treatment was obtained, including timing and duration for treatment, acupoint selection, types and method of acupuncture procedure;
(5) all the outcome measures and its intergroup differences.

If the data for meta-analysis were missing or only expressed graphically, we attempted to contact the authors to obtain the detailed data. Any disagreement noticed in the process of data cross-checking will be discussed with the third reviewer.(ZYJ).

#### 2.5.3 Assessment of risk of bias and reporting quality of included studies

We will evaluate the methodological quality of the included studies by using a checklist of the Collaborative Approach to Meta-Analysis and Review of Animal Data from Experimental Studies^[26]^ with minor modifications.^[27]^ Ten items are as follows:

(1) publication in a peer-reviewed journal;
(2) statement of temperature control;
(3) random allocation to groups;
(4) allocation concealment;
(5) blinded assessment of outcome;
(6) use of anesthetic without significant intrinsic cardioprotective activity;
(7) appropriate animal model (aged, diabetic, or hypertensive);
(8) sample size calculation;
(9) compliance with animal welfare regulations;
(10) statement of potential conflict of interests.

The assessment of risk of bias will be carried out by 2 independent reviewers (LZZ and ZXL) Any disagreements will be arbitrated by a third reviewer (ZYJ).

#### 2.5.4 Measurement of treatment effect

Efficacy data will be synthesized and statistically analyzed in RevMan 5.3 software. For dichotomous data, a risk ratio with 95% confidence intervals will be used for analysis. For continuous data, a mean difference or a standard mean difference with 95% confidence intervals will be used for analysis.

#### 2.5.5 Unit of analysis issues

If outcomes are presented from the studies of animals at different time points, we extracted data from the last time point. For studies comparing different intensity or frequency of acupuncture treatment to a single control group, the data of treatment group with the maximum amount of treatment will be selected for analysis.

#### 2.5.6 Dealing with missing data

The corresponding authors or relevant authors will be contacted to obtain insufficient data or missing data. If we received no response to our request, we will use GetData software to measure the data in the graph. If the complete data is still not available, then we will exclude the article of missing data from the data synthesis.

#### 2.5.7 Assessment of heterogeneity

Heterogeneity between studies results will be investigated based on *I*^2^ statistic. It will be considered significant heterogeneity while *I*^2^ > 50%. On the contrary, When *I*^2^ ≤50%, study will be regarded as little or no heterogeneity.

#### 2.5.8 Assessment of publication bias

A funnel plot analysis will be conducted to determine publication bias if 10 or more studies are included in the meta-analysis.

#### 2.5.9 Data synthesis

All analyses will be performed with Revman 5.3 software by the Cochrane Collaboration. Meta-analysis will be performed for outcome measures reported in more than 2 studies. In the present meta-analysis, we will use the random effects model rather than the fixed effects model because heterogeneity between multi-studies has to be taken into account. If the *I*^2^ test indicates unacceptable heterogeneity (*I*^2^ > 50%), sensitivity analysis and subgroup analysis will be performed to find out any possible reasons that may cause the heterogeneity. If meta-analysis is impossible, data will be reported by a descriptive summary. Probability value *P* < .05 is considered statistically significant.

#### 2.5.10 Subgroup analysis

Subgroup analyses on the following factors will be conducted to assess heterogeneity as well as possible: infarct time, reperfusion time, species of rat, type of acupuncture, acupoint prescription, the type of anesthetic, and the quality of study.

#### 2.5.11 Sensitivity analysis

After conducting a quality assessment of the included studies, we will conduct a sensitivity analysis if there are studies of low quality. Sensitivity analysis will also be performed when heterogeneity testing suggests unacceptable heterogeneity between studies (*I*^2^ > 50%). Then we will obtain a stable consolidated result of our study.

## 3 Discussion

Acupuncture pretreatment, as a safe and reliable measure, is derived from the thought of preventive treatment in TCM. Before the occurrence of the disease, using acupuncture stimulate certain acupuncture points or meridians in advance can increase the pre-adaptability of the body to diseases, thereby reducing subsequent damage of diseases to organs.^[17]^ At present, this intervention is widely used in health care and provides broad application prospects in the prevention and treatment of cardiovascular diseases. Additionally, Neiguan (PC6) acupoint has always been the preferred acupoint for the acupuncture therapy of cardiovascular diseases, which is located 2 Cun proximal to the wrist, between the flexor carpi radialis and the palmaris longus tendons.^[28]^ Hence we chose Neiguan (PC6)-based acupuncture pretreatment as the intervention in this review. The therapeutic strategy not only combines the triple function of acupuncture, Neiguan acupoint and pretreatment, but also provide new treatment ideas in improving MIRI. Especially in clinical practice, this simple therapy is anticipated to protect heart against MIRI before revascularization.

Systematic reviews of pre-clinical animal data could clarify the underlying mechanism of human diseases,^[29]^ preclude unnecessary study replication and improve the likelihood of success of future clinical trials.^[30]^ Therefore, this systematic review will provide an in-depth summary and latest analysis of the latest evidence on the efficacy of Neiguan (PC6)-based acupuncture pretreatment in animal models of MIRI. We expect the findings of this study would provide reference basis for Chinese guidelines on the treatment of MIRI as well as promote acupuncture treatment and application of acupuncture points so as to benefit more patients in the future.^[31]^

However, this systematic review also has potential limitations. Different types of acupuncture, needling techniques, and number of treatments may cause considerable heterogeneity in this review. In addition, limited to language ability, only studies in English and Chinese will be included, and reports in other languages may be ignored.

## Author contributions

Qiqi Yang and, Huifang Mao and Xia Chen have contributed equally to this work. All authors have read and approved the publication of the protocol

**Conceptualization:** Qiqi Yang, Huifang Mao, Xia Chen.

**Data curation:** Xia Chen, Xiaolei Zhang, Zhenzhen Liu.

**Formal analysis:** Huifang Mao, Gongdao Jiang, Wei Huang.

**Investigation:** Qiqi Yang, Yanji Zhang.

**Methodology:** Xiaolei Zhang, Gongdao Jiang, Wei Huang.

**Software:** Qiqi Yang, Yanji Zhang, Zhenzhen Liu.

**Supervision:** Gongdao Jiang, Wei Huang.

**Writing – original draft:** Qiqi Yang, Huifang Mao, Xia Chen, Yanji Zhang.

**Writing – review & editing:** Xiaolei Zhang, Zhenzhen Liu, Gongdao Jiang, Wei Huang.
